# Incidence and predictors of preterm neonatal mortality at public hospitals in Eastern Ethiopia: A prospective follow-up study

**DOI:** 10.1371/journal.pone.0327766

**Published:** 2025-08-01

**Authors:** Teshale Mengesha, Tsegasew Embiale, Dawit Melese, Natnael Dechasa Gemeda, Mickiale Hailu, Aminu Mohamed, Alemu Guta, Yomilan Geneti, Muluken Yigezu, Tewodros Getnet Amera

**Affiliations:** 1 Department of Pediatrics and Child Health Nursing, College of Medicine and Health Sciences, Dire Dawa University, Dire Dawa, Ethiopia; 2 Dire Dawa University, College of Medicine and Health Sciences, Department of Midwifery, Dire Dawa, Ethiopia; 3 Department of Public Health, College of Medicine and Health Sciences, Dire Dawa University, Dire Dawa, Ethiopia; JSI, ETHIOPIA

## Abstract

**Introduction:**

Preterm birth is a major clinical problem, affecting 15 million births every year worldwide. It is the largest cause of death in children under the age of five. Even though different studies have been done regarding the incidence of preterm neonatal death in some parts of Ethiopia, there is a paucity of evidence, especially in the eastern part. Therefore, this study aimed to assess the incidence and predictors of preterm neonatal mortality at public hospitals in eastern Ethiopia from May 1, 2022, to March 30, 2023.

**Methods:**

An institution-based prospective follow-up study was conducted at public health institutions from May 1, 2022, to March 30, 2023. A structured, pretested, and interviewer-administered questionnaire and checklist were used to collect the data. The data were entered into EpiData version 4.6 and then exported to STATA version 14 for analysis. Kaplan-Meier was used to estimate survival time. A log-rank test was used to compare survival curves between different categories of the explanatory variables. Descriptive analysis, bivariable, and multivariable Cox Proportional Hazards regression analysis were performed. The level of significance was declared at a p-value of <0.05.

**Results:**

The overall incidence rate of death was 17.03 (14.3, 20.28) per 1000 person-day observations. Mothers’ obstetric complications (AHR: 2.27, 95% CI: (1.46, 3.54)), not crying at birth (AHR: 1.83, 95% CI: (1.09, 2.31)), not immediately initiating breastfeeding (HR: 1.59, 95% CI: (1.10, 2.31)), having sepsis (HR: 1.85, 95% CI: (1.26, 2.72)), having respiratory distress syndrome (HR: 1.98, 95% CI: (1.36, 2.90)), having perinatal asphyxia (HR: 3.1, 95% CI: (2.13, 4.51)), and an APGAR score of less than seven (HR: 2.16, 95% CI: (1.47, 3.16)) were significant predictors of preterm neonatal death.

**Conclusion:**

This study found the high incidence rate of preterm neonatal mortality, primarily driven by preventable and manageable conditions. Strengthening early identification and timely intervention for the risk factors is critical to improving survival outcomes among preterm neonates.

## Background

Preterm birth is a major clinical problem, affecting 15 million births every year worldwide. It is the largest cause of death in children under the age of five, with the bulk of deaths happening during the neonatal period [1]. Globally, about 2.4 million children died in the first month of life in 2019, which equates to nearly 6,700 neonatal deaths every day. Preterm babies born in developing countries face higher morbidity and mortality rates compared to those in developed countries [2]. Sub-Saharan Africa (SSA) has the highest neonatal mortality rate. A child born in SSA is 10 times more likely to die in the first month than a child born in a high-income country [3].

A study in Jordan showed that the neonatal mortality rate was 30 times higher in preterm neonates than in term neonates, indicating a survival gap between the two groups [4]. The incidence of preterm neonatal mortality observed in Uganda was high, especially among very preterm neonates, with the majority of deaths occurring in the early neonatal period [5]. Similarly, a study in Nigeria indicated that the early neonatal mortality rate for preterm babies was high, which may reflect suboptimal prenatal and newborn care [6].

In Ethiopia, according to the 2019 mini Ethiopian Demographic Health Survey (EDHS), neonatal mortality has been reported as 30/1000 live births [7]. Studies in Ethiopia have revealed that the incidence of preterm neonatal mortality has ranged from 29.2 to 62.1 deaths per 1000 person-days of follow-up [8,9]. Another prospective follow-up study revealed in eastern Ethiopia, the proportion of neonatal mortality was unacceptably high (20%), primarily caused by preterm birth complications [10]. A retrospective study at Dil Chora Hospital in Dire Dawa, Ethiopia, reported a neonatal mortality rate of 11.44% with prematurity being the leading cause of admission [11].

Despite improving neonatal health being a government priority, with various strategies and policies designed to reduce neonatal mortality, the desired outcomes have not been achieved.

There are different recent efforts and recommendations both nationally and internationally to reduce preterm neonatal deaths. These include support for the implementation of neonatal policies and plans, strengthening programs to deliver low-cost, high-impact interventions [12], provision of appropriate antenatal care by trained healthcare providers, early initiation of feeding, and better referral linkages [12,13]

Despite applying these preventive approaches, several risk factors increase the incidence of mortality among preterm neonates. These factors include a history of previous neonatal death, the need for cardiopulmonary resuscitation (CPR), presence of anomalies, Apgar score <7, multiple pregnancies, non-cephalic presentation, low birth weight, PNA, necrotizing enterocolitis (NEC), RDS, and hypothermia [14,15].

To achieve the Sustainable Development Goal (SDG) target 3, which focuses on ending preventable deaths of newborns and aims to reduce neonatal mortality to at least as low as 12 per 1,000 live births by 2030 [16], up-to-date information regarding the occurrence of death and its predictors among preterm neonates is very important. Studies in Ethiopia strongly recommend further longitudinal prospective follow-up studies to identify additional factors that determine preterm survival [8,17,18]. Even though different studies have been conducted regarding the incidence of preterm neonatal death in some parts of our country, all were conducted retrospectively. Additionally, there is no study specifically on preterm mortality in the eastern part of our country. Therefore, the aim of this study is to assess the incidence and predictors of preterm neonatal mortality at public hospitals in the study area.

The information generated through this study offers knowledge for health professionals in the early identification of high-risk neonates and timely intervention for their better survival.

## Methods

### Study design, area and period

A prospective follow-up study was conducted in public hospitals in eastern Ethiopia from May 1, 2022, to March 30, 2023. Harari region and Dire Dawa city Administration were purposively selected as study areas. Two hospitals from the Harari Region and two hospitals from Dire Dawa city were selected. Harar is the capital city of the Harari region and the east Hararghe Zone of Oromia region. Harar is located 526 kilometers from the capital city, Addis Ababa. According to the 2007 Ethiopian census projection for 2019/2020, the current estimated total population of the city is 263,656.

There are 2 public hospitals, 2 private hospitals, 1 police hospital, 8 health centers (4 urban and 4 rural), 54 private clinics, and 24 health posts in the Harari Region. Dire Dawa city is one of the two self-administered cities in Ethiopia and is located 515 kilometers east of the capital city, Addis Ababa, and 47 kilometers from Harar. According to the 2007 Ethiopian census projection for 2019/2020, the current estimated total population of the city is 492,637. There are 2 public hospitals, 4 private hospitals, 15 health centers (8 urban and 7 rural), and 32 health posts under the Dire Dawa City administration.

### Source population

All preterm neonates who were admitted to the neonatal intensive care unit (NICU).

#### Study population.

All preterm neonates who were admitted to the NICU from May 1, 2022, to March 30, 2023.

#### Inclusion criteria.

All live preterm neonates born at a gestational age from 28 weeks to less than 37 weeks during the study period were included. However, all preterm neonates with unknown gestational age and those admitted without their mothers were excluded.

### Sample size determination and sampling procedure

The sample size was determined using the double population proportion formula for a cohort study, and it was calculated using the Log-rank test (Freedman method), a two-sample comparison of survival functions, through the STATA version 14.0 software. Significantly associated predictors (RDS, PNA, congenital malformation) of mortality among preterm neonates from the study conducted in Debre Markos, Ethiopia, were used, assuming a 95% confidence interval, a power of 90%, and a one-to-one ratio of exposed to unexposed groups [8]. The final sample size was determined to be 478 by selecting the larger sample size.

After obtaining the average number of preterm monthly admissions in the NICU from each hospital, the sample size was proportionally allocated using the formula ni = Ni(n/N). Then, all live preterm neonates that were admitted during the study period were consecutively enrolled in the study.

### Data collection tools and procedures

A structured questionnaire and checklist were adapted from a review of related literature [5,8,17] and national neonatal and delivery registration books. An appropriate data extraction format was prepared in English, which needed to be translated into four local languages (Amharic, Afan Oromo, Somali, and Adere). The data extraction form included socio-demographic characteristics, maternal medical and obstetrics-related characteristics, and neonatal-related information. Mothers of the eligible preterm neonates were counseled before enrollment and after delivery. Those who provided written informed consent were subsequently enrolled alongside their neonates. The interviewer administered the questionnaire to obtain baseline data from the mothers and followed-up with a chart review to obtain additional information about the mothers. Preterm neonates subsequently admitted to the NICU were continuously followed-up to ascertain outcomes through observation and chart review from admission time to discharge or death, up to a maximum of 28 days of life. The data collectors conducted a daily follow-up of the neonates in the NICU to identify if they had been discharged, died, referred, or lost to follow-up. For any preterm neonate discharged alive before 28 days, post-discharge follow-up was conducted via phone calls on days 3, 7, 14, and 28.

### Study variables

#### Dependent variables.

Incidence of death

#### Independent variables.

***Socio-demographic related predictors.*** Maternal age, level of education, marital status, occupation, residence, referral status, sex of the neonate, place of birth

***Maternal medical and obstetrics related predictors.*** Parity, gestational age at first booking ANC visit, gestational age at birth, number of ANC visits, antenatal corticosteroid (ACS) administration, mode of delivery, prior preterm birth, prior early neonatal death, prolonged premature rupture of membrane (PPROM), multiple pregnancy, preeclampsia/eclampsia, placenta previa or abruption placenta, and chorioamnionitis, diabetes mellitus (DM), HIV/AIDS, anemia.

***Neonatal related predictors.*** Birth weight of neonate, APGAR score at 5^th^ minutes, congenital anomalies, congenital heart disease, perinatal asphyxia, respiratory distress syndrome, hypothermia, hypoglycemia, necrotizing enterocolitis, sepsis, jaundice, breastfeeding (BF), kangaroo mother care (KMC), cry at birth, oxygen therapy, phototherapy, resuscitation.

### Operational definition

***Preterm neonate***: all live births before 37 completed weeks (whether singleton or multiple)

***Event***: the occurrence of death during the follow-up time.

***Censored***: those who withdraw from study, lost to follow-up, live at 28 days, and those referred to other health institution.

***Survival status***: outcome of preterm neonates either died or censored.

***Obstetric complication during current pregnancy***: if the mother had one or more of the following complications such as preeclampsia/eclampsia, PPROM, antepartum hemorrhage (APH), chorioamnionitis.

### Data quality control

Training was provided to data collectors by the principal and co-investigators before the actual data collection period. A pretest was performed on 5% of the sample size one month before the actual data collection time at Dil Chora Hospital. The data were collected by four nurses assigned to the NICU of the selected hospitals. The principal investigator and co-investigator continuously supervised the data collectors, and the data were checked for completeness and consistency by the investigators. Finally, the data were stored in a secure location where only the investigators could access it.

### Data processing and analysis

After completeness and consistency were checked, the collected data were coded and entered into EPI Data version 4.6. Then, it was exported to STATA version 14 for analysis. The assumption of the Cox Proportional Hazards regression model was checked by running a global test based on the scaled Schoenfeld residuals test. Multicollinearity was assessed using a variance inflation factor and correlation coefficient. Descriptive statistics were computed as frequencies and percentages, and the results were displayed using tables, charts, and graphs. Outcomes of each participant were dichotomized into censored or events. The incidence rate was determined using person-days of follow-up as a denominator for the entire cohort. Kaplan-Meier analysis was used to estimate survival time and cumulative probability of survival. The Log-rank test was used to compare survival curves between different categories of the explanatory variables. The bivariable Cox Proportional Hazards model was employed to check variables that have a P-value < 0.2. Then, variables with this value were selected for the multivariable Cox Proportional Hazard regression model. A hazard ratio with a 95% confidence interval and a P-value < 0.05 was used to measure the association and to consider statistically significant risk factors for time to death.

### Ethics approval and consent to participate

Ethical clearance was obtained from the Institutional Review Board of Dire Dawa University (protocol number DDU-IRB-2022–069 and date of issued: 28 Feb 2022). Following ethical clearance, official letters of cooperation were obtained from the Dire Dawa City administration health office and the Harari regional health bureau to facilitate the research. Permission was obtained from each head of the health institution. Written informed consent was obtained from the literate mothers of the neonates and the written consent was explained for illiterate groups by data collectors, and then signed by their fingerprint. Confidentiality of the information was maintained throughout the data collection process. The study adhered to the relevant guidelines and principles of the Declaration of Helsinki.

## Results

### Socio-demographic predictors of the mother and the neonate

In this study, 478 preterm neonates were followed from May 1, 2022, to March 30, 2023. Among those preterm neonates, slightly more than half, 257 (53.77%), were female. Nearly half, 234 (48.95%), of the preterm neonates were delivered at a gestational age of less than 34 weeks. The median gestational age of the participants at delivery was 33 weeks (IQR = 31, 34). About half, 243 (50.84%), of the participants were delivered by mothers aged 25–34 years. The highest proportions of deaths, 74 (58.73%) and 75 (59.52%), occurred among preterm neonates residing in rural areas and those referred from other facilities, respectively (Table 1).

**Table 1:** Socio-demographic characteristics of preterm neonates at public hospitals in eastern Ethiopia, 2023 (n = 478).

Variables	Categories	N = 478 (%)	Outcome	
			Event (%)	Censored (%)
Maternal age	<18	37(7.74)	17(13.49)	20(5.68)
	19-24	146(30.54)	46(36.51)	100(28.41)
	25-34	243(50.84)	43(34.13)	200(56.82)
	≥35	52(10.88)	20(15.87)	32(9.09)
Educational status	Unable to read and write	124(25.94)	48(38.10)	76(21.59)
	Primary school	161(33.68)	48(38.10)	113(32.10)
	Secondary	116(24.27)	11(8.73)	105(29.83)
	College/university	77(16.11)	19(15.08)	58(16.48)
Marital status	Single	21(4.39)	5(3.97)	16(4.55)
	Married	441(92.26)	115(91.27)	326(92.61)
	Divorced	7(1.46)	2(1.59)	5(1.42)
	Widowed	2(0.42)	2(1.59)	0(0.00)
	Separated	7(1.46)	2(1.59)	5(1.42)
Occupation	Employed	101(21.13)	24(19.05)	77(21.88)
	Merchant	110(23.01)	17(13.49)	93(26.42)
	Farmer	173(36.19)	52(41.27)	121(34.38)
	Housewife	89(18.62)	30(23.81)	59(16.76)
	Student	5(1.05)	3(2.38)	2(0.57)
Sex of the neonate	Male	221(46.23)	58(46.03)	168(46.31)
	Female	257(53.77)	68(53.97)	189(53.69)
Residency	Urban	244(51.05)	52(41.27)	192(54.55)
	Rural	234(48.95)	74(58.73)	160(45.45)
Referral status	Referred in	231(48.33)	75(59.52)	156(44.32)
	Not referred in	247(51.67)	51(40.48)	196(55.68)

### Maternal related predictors

The highest proportion of events, 76 (60.31%), occurred among preterm neonates of mothers having parity less than two. Most (91.0%, or 435) preterm neonates were delivered by mothers who had received antenatal care. The highest percentage, 406 (84.94%), of neonates’ mothers received antenatal corticosteroids. More than sixty-five percent of preterm neonates were delivered by spontaneous vaginal delivery. Approximately three-fourths (75.40%, or 95) of deaths occurred among neonates of mothers experiencing obstetric complications, including preeclampsia/eclampsia, preterm premature rupture of membranes, antepartum hemorrhage, and other factors (Table 2).

**Table 2:** Maternal related information of preterm neonates at public hospitals in eastern Ethiopia, 2023 (n = 478).

Variables	Categories	N = 478 (%)	Outcome	
			Event (%)	Censored (%)
Gestational age at birth	<34	234(48.95)	79(62.70)	155(44.03)
	≥34	244(51.05)	47(37.30)	197(55.97)
Number of parity	Primipara	257(53.77)	76(60.31)	181(51.42)
	Multipara	221(46.23)	50(39.68)	171(48.57)
Antenatal follow up	No	43(9.00)	25(11.9)	28(7.95)
	Yes	435(91.00)	111(88.09)	324(92.04)
Number of ANC visits	No visit	52(10.88)	15(11.90)	28(7.95)
	1-2	194(40.59)	55(43.65)	139(39.49)
	3-4	231(48.33)	51(40.47)	184(52.27)
	>4	1(0.21)	5(3.96)	1(0.28)
Received ACS before delivery	No	72(15.06)	14(11.11)	58(16.48)
	Yes	406(84.94)	112(88.89)	294(83.52)
Status of pregnancy	Single	380(79.50)	86(68.25)	294(83.52)
	Twin	88(18.41)	33(26.19)	55(15.63)
	Multiple	10(2.09)	7(5.56)	3(0.85)
Mode delivery	Spontaneous	315(65.90)	75(59.52)	240(68.18)
	Cesarean section	152(31.80)	51(40.48)	101(28.69)
	Vaginal Breach	5(1.05)	0(0.00)	5(1.42)
	Instrumental delivery	6(1.26)	0(0.00)	6(1.70)
Having obstetric complications	Yes	241(50.42)	95(75.40)	146(41.48)
	No	237(49.58)	31(24.60)	206(58.52)
Previous history of preterm delivery	Yes	41(8.58)	21(16.67)	20(5.68)
	No	437(91.42)	105(83.33)	332(94.32)
History of early neonatal death	Yes	54(11.30)	34(26.98)	20(5.68)
	No	424(88.70)	92(73.02)	332(94.32)
Having medical problem during pregnancy	Yes	47(9.83)	16(12.70)	31(8.81)
	No	431(90.17)	110(87.30)	321(91.19)

Abbreviations: ANC; Antenatal Care, ACS; Antenatal corticosteroid

### Neonatal related predictors

More than half (54.76%, or 69) of the events or deaths occurred among participants who did not cry immediately at birth. Slightly more than half (51.59%, or 65) of the events occurred among preterm neonates who did not breastfeed immediately. Approximately 162 (33.89%) of the participants were diagnosed with respiratory distress syndrome, 157 (32.85%) with sepsis, and 119 (24.90%) with perinatal asphyxia at admission, respectively (Table 3).

**Table 3:** Neonatal and intervention related information of preterm neonates at public hospitals in eastern Ethiopia, 2023 (n = 478).

Variables	Categories	N = 478 (%)	Outcome	
			Death (%)	Censored (%)
Place of birth	Health facility	457(95.61)	117(92.86)	340(96.59)
	Out of health facility	21(4.39)	9(7.14)	12(3.41)
Birth weight	≤2000gm	381(79.71)	113(89.68)	268(76.14)
	>2000gm	97(20.29)	13(10.32)	84(23.86)
Crying at birth	No	172(35.98)	69(54.76)	103(29.26)
	Yes	306(64.02)	57(45.24)	249(70.74)
APGAR score at 5^th^ minute	<7	219(45.82)	77(61.11)	142(40.34)
	≥7	259(54.18)	49(38.89)	210(59.66)
Breast feeding initiated immediately	Yes	164(34.31)	61(48.41)	103(29.26)
	No	314(65.69)	65(51.59)	249(70.74)
Sepsis	Yes	157(32.85)	72(57.14)	85(24.15)
	No	321(67.15)	54(42.86)	267(75.85)
Jaundice	Yes	60(12.55)	33(26.19)	27(7.67)
	No	418(87.45)	93(73.81)	325(92.33)
RDS	Yes	162(33.89)	72(57.14)	90(25.57)
	No	316(66.11)	54 (42.86)	262(74.43)
Hypothermia	Yes	109(22.80)	13(10.32)	96(27.27)
	No	369(77.20)	113(89.68)	256(72.73)
Hypoglycemia	Yes	32(6.69)	8(6.35)	24(6.82)
	No	446(93.31)	118(93.65)	328(93.18)
CHD	Yes	17(3.56)	7(5.56)	10(2.84)
	No	461(96.44)	119(94.44)	342(97.16)
NEC	Yes	5(1.05)	3(2.38)	2(0.57)
	No	473(98.95)	123(97.62)	350(99.43)
PNA	Yes	119(24.90)	66(52.38)	53(15.06)
	No	359(75.10)	60(47.62)	299(84.94)
KMC applied	No	275(57.53)	58(46.03)	14(41.19)
	Yes	203(42.47)	68(53.97)	207(58.81)
Oxygen therapy given	No	321(67.15)	68(53.97)	253(71.88)
	Yes	157(32.85)	58(46.03)	99(28.13)
Ventilated	No	205(42.89)	57(45.23)	148(42.04)
	Yes	273(57.11)	69(54.76)	204(57.95)
Phototherapy given	No	134(28.03)	20(15.87)	114(32.39)
	Yes	344(71.97)	106(84.13)	238(67.61)
NGT feeding given	No	211(44.14)	35(27.78)	176(50.00)
	Yes	267(55.86)	91(72.22)	176(50.00)

Abbreviations: RDS; Respiratory Distress Syndrome, CHF; Congenital Heart Disease, PNA;Perinatal Asphyxia, KMC; Kangaroo Mother Care, NGT; Nasogastric Tube

### Incidence of death during follow-up

The study participants were followed for a minimum of one day and a maximum of 28 days. The total observation period for the follow-up was 7398 neonate days. Of the 478 study participants, 126 (26.36%) died, while the remaining 352 (73.64%) were censored. This study found that the cumulative incidence of mortality among preterm neonates was 26 per 100 live births. The incidence density was 17.03 (14.3, 20.28) per 1000 person-days of observation.

### The survival status of preterm neonates

**Fig 1:**
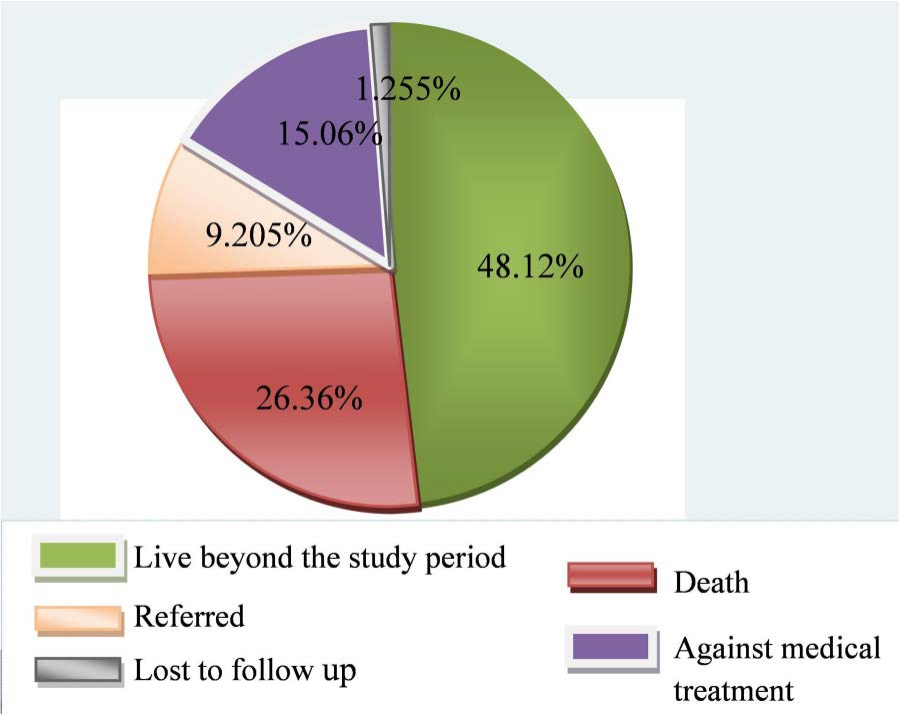
Outcome status of preterm neonates at public hospitals in Harari region and Dire Dawa city, eastern Ethiopia from May 1, 2022 to March 30, 2023(n= 478).

**Fig 2:**
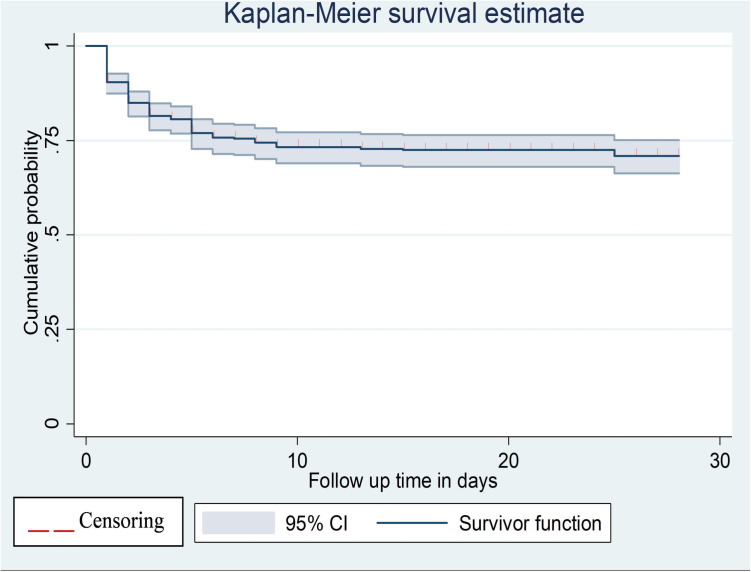
The overall Kaplan-Meier curve of survival probability of preterm neonates at public hospitals in eastern Ethiopia, 2023 (n = 478).

Of all participants followed, about 48.12%, 26.36%, and 15.06% were alive at the end of the follow-up, died, and left against medical advice, respectively (Fig 1). The study found that the overall survival probability among preterm neonates remained 71% (66.30, 75.06), indicating survival above the median (Fig 2). The cumulative probability of survival was 78.6% (74.71, 8.20) from the first to the third days, 62.3% (57.83, 66.52) from the third to the seventh days, and 29.9% (25.87, 34.06) from the seventh to the 28th days, respectively.

### Survival time of preterm neonates among categories of variables

**Fig 3:**
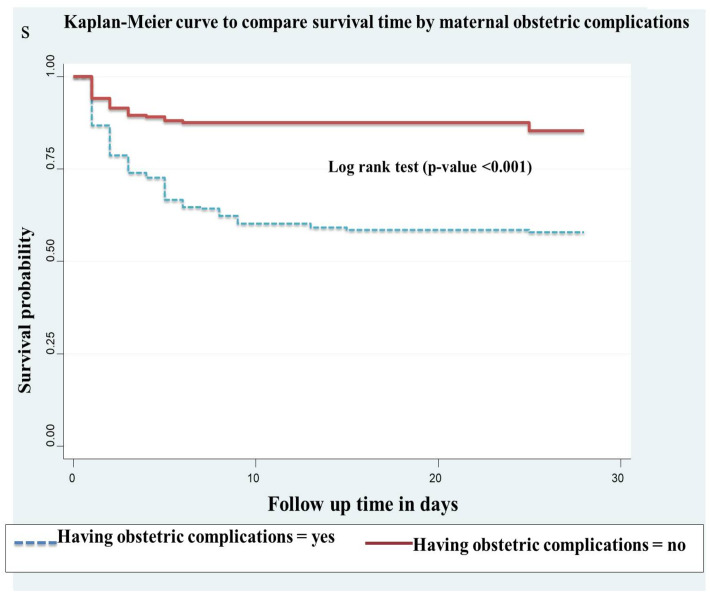
Kaplan-Meier curve to compare survival time of preterm neonates with categories of maternal obstetric complications at public hospitals in Harari region and Dire Dawa city, eastern Ethiopia from May 1, 2022 to March 30, 2023(n = 478).

In this study, preterm neonates whose mothers experienced obstetric complications had shorter survival times compared to preterm neonates whose mothers did not have complications. The cumulative survival probabilities of these two groups were 24% and 35.8%, respectively. This difference was statistically significant (p-value <0.001) (Fig 3).

**Fig 4:**
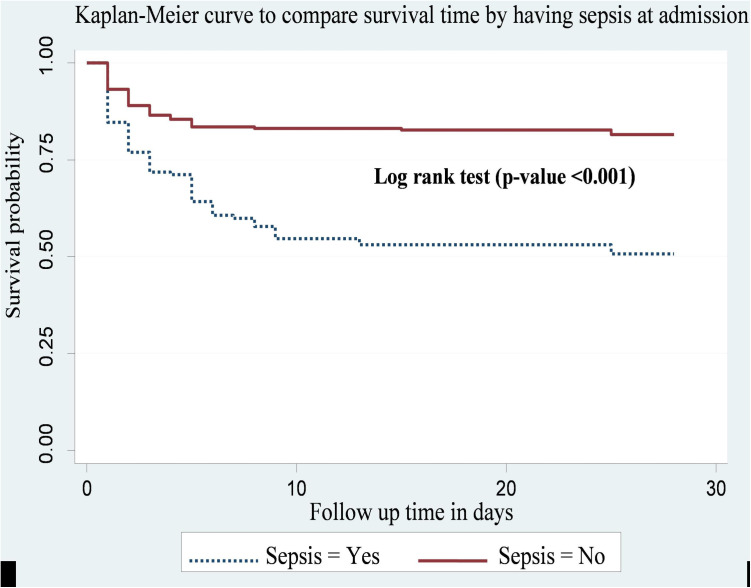
Kaplan-Meier curve to compare survival time of preterm neonates with categories of sepsis at public hospitals in Harari region and Dire Dawa city, eastern Ethiopia from May 1, 2022 to March 30, 2023(n = 478).

Preterm neonates diagnosed with neonatal sepsis at admission had shorter survival times compared to those without sepsis. The cumulative survival probabilities of preterm neonates with sepsis and without sepsis were 19.1% and 35.2%, respectively. This difference was statistically significant (p-value <0.001) (Fig 4).

**Fig 5:**
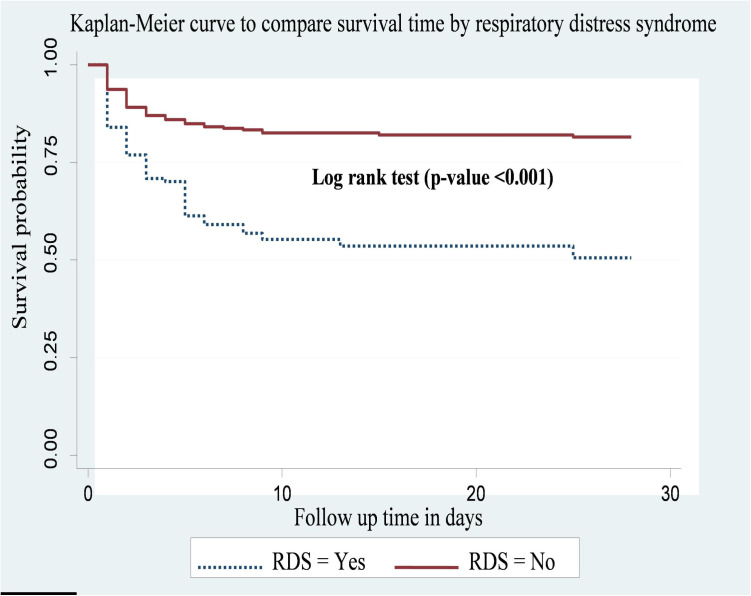
Kaplan-Meier curve to compare survival time of preterm neonates with categories of RDS at public hospitals in Harari region and Dire Dawa city, eastern Ethiopia from May 1, 2022 to March 30, 2023(n = 478).

Preterm neonates diagnosed with respiratory distress syndrome (RDS) at admission had shorter survival times compared to those without RDS. The overall survival probabilities of preterm neonates with RDS and without RDS were found to be 23.4% and 33.2%, respectively. This difference was statistically significant by log-rank test (p-value <0.001) (Fig 5).

**Fig 6:**
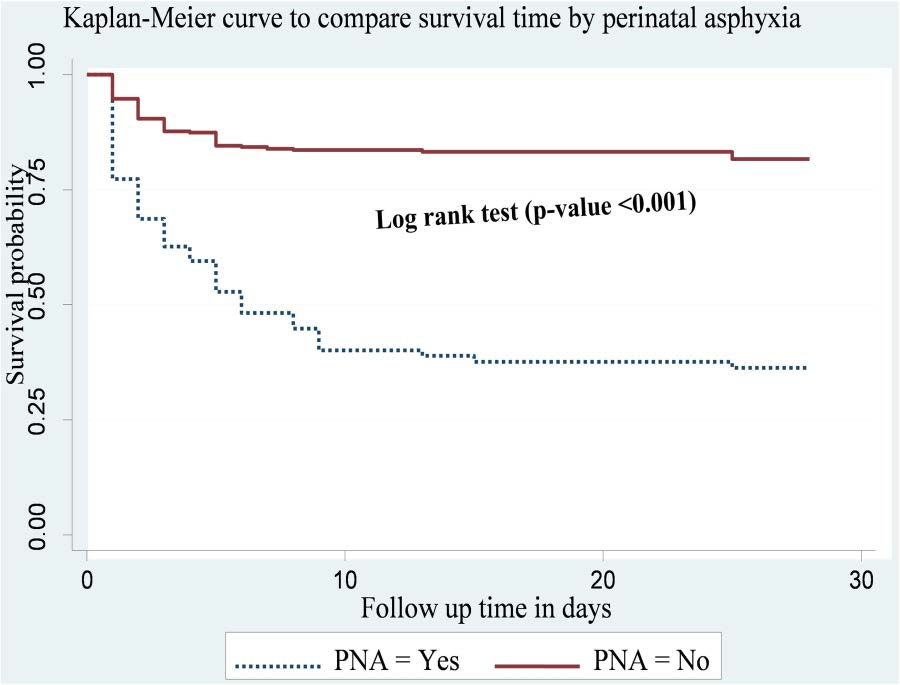
Kaplan-Meier curve to compare survival time of preterm neonates with categories of PNA at public hospitals in Harari region and Dire Dawa city, eastern Ethiopia from May 1, 2022 to March 30, 2023(n = 478).

Preterm neonates with PNA had shorter survival times compared to those without PNA. The cumulative survival probability of neonates with PNA and without PNA at the end of follow-up was 17.6% and 34%, respectively. This difference was statistically significant (p-value <0.001) (Fig 6).

### Predictors of death among preterm neonates

The Cox Proportional Hazard model was used to identify predictors of death among preterm neonates. In bivariate Cox regression, variables eligible for multivariable Cox regression at a p-value less than 0.25 included: maternal obstetric complication, history of early neonatal death, crying at birth, referral status, breastfeeding initiated immediately, ventilation, sepsis, jaundice, RDS, hypothermia, PNA, APGAR score at the 5th minute, previous history of preterm delivery, number of parity, and antenatal follow-up. Multivariable analysis identified maternal obstetric complication, crying at birth, breastfeeding initiated immediately, sepsis, RDS, PNA, and APGAR score at the 5th minute as significant predictors of mortality among preterm neonates.

The hazard of death among preterm neonates whose mothers had obstetric complications was 2.27 times higher (AHR: 2.27, 95% CI: 1.46–3.54) compared to preterm neonates whose mothers did not have complications. Preterm neonates who did not cry immediately after birth had an 83% increased hazard of death (AHR: 1.83, 95% CI: 1.09–2.31) compared to those who did cry immediately after birth. Preterm neonates who did not immediately initiate breastfeeding had a 59% increased hazard of death (HR: 1.59, 95% CI: 1.10–2.31) compared to those who did. Preterm neonates diagnosed with sepsis had an 85% increased risk of death (HR: 1.85, 95% CI: 1.26–2.72) compared to those without sepsis at admission. Similarly, preterm neonates diagnosed with RDS had a 98% increased risk of death (HR: 1.98, 95% CI: 1.36–2.90) compared to those without RDS. Preterm neonates diagnosed with PNA were 3 times more likely to die (HR: 3.1, 95% CI: 2.13–4.51) compared to those without PNA. The hazard of death for preterm neonates with an APGAR score less than seven was about 2 times higher (HR: 2.16, 95% CI: 1.47–3.16) compared to those with an APGAR score greater than seven (Table 4).

**Table 4:** Bivariable and multivariable Cox Proportional Hazard regression analysis of predictors of death among preterm neonates at public hospitals in Eastern Ethiopia, 2023 (n = 478).

Variable	Category	CHR(95% CI)	AHR(95% CI)	P-value
Having obstetric complications	Yes	3.28(2.18, 4.92)	2.27(1.46, 3.54)	0.000
	No	1	1	
History of early neonatal death	Yes	3.37(2.27, 5.00)	1.25(0.74, 2.10)	0.392
	No	1	1	
Crying at birth	No	2.30(1.62, 3.27)	1.83(1.27, 2.65)	0.001
	Yes	1	1	
Referral status	Referred in	1.53(1.07, 2.18)	1.43(.97, 2.11)	0.067
	Not referred in	1	1	
Breast feeding initiated immediately	No	1.80(1.27, 2.56)	1.59(1.09, 2.31)	0.014
	Yes	1	1	
Ventilated	No	1.24(.87, 1.76)	1.31(0.88, 1.95)	0.173
	Yes	1	1	
Sepsis	Yes	2.94(2.06, 4.18)	1.85(1.26, 2.72)	0.002
	No	1	1	
Jaundice	Yes	2.79(1.87, 4.16)	1.01(.63, 1.63)	0.940
	No	1	1	
RDS	Yes	2.96(2.08, 4.22)	1.95(1.34, 2.85)	0.000
	No	1	1	
Hypothermia	Yes	0.33(0.18, 0.59)	0.76(.41, 1.413)	0.398
	No	1	1	
PNA	Yes	4.40(3.09, 6.26)	3.1(2.13, 4.51)	0.000
	No		1	
APGAR score at 5th minute	<7	2.13(1.48, 3.04)	2.16(1.47, 3.16)	0.000
	≥7	1	1	
Previous history of preterm delivery	Yes	2.21(1.38, 3.53)	1.08(.58, 2.11)	0.739
	No	1	1	
Number of parity	<2	0.34 (0.19, 0.61)	0.78(0.43, 1.51)	0.289
	≥2	1	1	
Antenatal follow-up	No	1.31(0.76, 2.24)	1.55(0.88, 2.76)	0.128
	Yes	1	1	

Abbreviations: CHR: Crude Hazard ratio, AHR: Adjusted Hazard Ratio, CI: Confidence Interval, PNA: Perinatal Asphyxia, RDS: Respiratory distress syndrome

## Discussion

This prospective study assessed the incidence and predictors of preterm neonatal mortality at public hospitals in the Harari region and Dire Dawa city. The cumulative incidence of preterm neonatal death was 26 per 100 live births. This was lower than the cumulative incidence reported in studies conducted in Gondar and Bahir Dar, which found rates of 28 and 31 per 100 live births, respectively [19,20]. The overall incidence of preterm neonatal mortality was 17.03 (14.3–20.28) per 1000 person-days of observation. This finding was lower than the incidence of mortality reported in studies conducted in Bahir Dar, Debre Markos, Tikur Anbesa, and Mizan Tepi, which found rates of 35, 29.2, 39.1, and 62.15 deaths per 1000 person-days of observation, respectively [8,17]. These variations might be due to the use of retrospective data, single study settings, larger sample sizes, and earlier study periods in previous studies. Similarly, this result was lower than the preterm mortality rates reported in studies conducted in Tanzania and Nigeria, which found rates of 39 and 24 per 100 live births, respectively [21,22]. These lower rates could be attributed to recent improvements in well-equipped newborn care facilities and the role of trained health professionals, which may have improved outcomes for preterm newborns [23].

This study found that preterm neonates whose mothers had obstetric complications had a higher hazard of mortality compared to those whose mothers did not have complications. This finding is consistent with a study conducted in Trinidad and Tobago [24]. These complications can lead to intrauterine growth restriction, maternal health deterioration, and neonatal sepsis, all of which can contribute to preterm neonatal mortality [25].

Preterm neonates who did not cry at birth had a higher hazard of death than those who did cry. This finding is supported by studies conducted in Gondar and Mizan Tepi [17,19]. Preterm neonates who did not cry immediately may have abnormal breathing patterns, which can be a sign of PNA, a leading cause of neonatal death [26].

This study found that preterm neonates who did not immediately initiate breastfeeding had an increased hazard of mortality compared to those who did. This finding is consistent with studies conducted in Bahir Dar and Uganda [5,20]. Early breastfeeding minimizes the risk of infection-related death by limiting the consumption of pathogenic microorganisms and providing a variety of immunocompetent elements that may activate the immune systems of neonates [27].

Preterm neonates diagnosed with sepsis, RDS, or PNA at admission had an increased risk of mortality. Similar findings have been reported in other studies [17,18,20,28]. Preterm neonates are vulnerable to sepsis due to deficient immune systems, immature epithelial barriers, and the increased need for invasive devices [8,18,20,28]. Respiratory distress syndrome (RDS) is a disease that causes newborns respiratory failure caused by prematurity-related surfactant deficiency and possesses a chance of lung collapse, which could result in hypoxia and, ultimately, death [18,29]. PNA causes decreased oxygenation and blood flow to cells or organs leading to neonatal brain injury, morbidity, and mortality.

This study also found that the hazard of death for preterm neonates with an APGAR score less than seven was about 2 times higher (HR: 2.16, 95% CI: 1.47–3.16) compared to those with an APGAR score greater than seven (Table 4). This is similar to what was reported in other studies [18,29]. The possible reason was poor 5th-minute APGAR score in preterm infants may be a sign of physiologic immaturity as well as an indication of infections, neurological conditions, and congenital defects that raise the possibility of death [30]. In contrast to previous studies that identified antenatal care (ANC) follow-up as a protective factor against preterm neonatal death, our study did not find a statistically significant association between ANC attendance and neonatal outcomes. This discrepancy may be due to differences in the quality of ANC visits, which were not fully captured in our dataset. It is also possible that limited variability in ANC attendance among participants may have influenced the findings. Despite this, the established importance of ANC in improving maternal and neonatal health remains well supported in the literature, and further research is needed to explore the specific components and quality of ANC that contribute most to neonatal survival.

### Limitation

The limitation of this study was its small sample size and the study did not start the follow-up time from the delivery room. Parents might give inaccurate reports of the baby’s condition during phone call follow-up. In addition, the nutritional status of the mother was not included which may be a significant predictor of mortality among preterm neonates.

## Conclusion

This study found that the incidence rate of preterm neonatal mortality was high. Variables such as maternal obstetric complications, not crying at birth, not initiating immediate breastfeeding, having sepsis, RDS, PNA, and APGAR score at the 5th minute were predictors of preterm neonatal death. Everyone should emphasize initiating immediate breastfeeding, surfactant provision, and proper management of sepsis and perinatal asphyxia.

## Supporting information

S1 Data Predictors of preterm neonatal death data. (DTA)

## Abbreviations:

AHR: Adjusted hazard ratio
ANC: Antenatal care
APGAR: Activity, pulse, grimace, appearance & respiration
BF: Breast feeding
EDHS: Ethiopian demographic health survey
GA: Gestational age
KM: Kaplan meier
KMC: Kangaroo mother care
LBW: Low birth weight
NICU: Neonatal intensive care unit
PH: Proportional hazard
PNA: Perinatal asphyxia
PY: Person year
SDG: Sustainable development goal
RDS: Respiratory distress syndrome
SSA: Sub-Saharan Africa
WHO: World health organization.

## References

[1] Walani SR. Global burden of preterm birth. Int J Gynaecol Obstet. 2020;150(1):31–3. doi: 10.1002/ijgo.13195 32524596

[2] WHO. Newborns: improving survival and well-being. 2020.

[3] Neonatal mortality. https://data.unicef.org/topic/child-survival/neonatal-mortality/. 2020. 2021 December 4.

[4] Abdel Razeq NM, Khader YS, Batieha AM. The incidence, risk factors, and mortality of preterm neonates: A prospective study from Jordan (2012-2013). Turk J Obstet Gynecol. 2017;14(1):28–36. doi: 10.4274/tjod.62582 28913132 PMC5558315

[5] Tibaijuka L, Bawakanya SM, Owaraganise A, Kyasimire L, Kumbakumba E, Boatin AA, et al. Incidence and predictors of preterm neonatal mortality at Mbarara Regional Referral Hospital in South Western Uganda. PLoS One. 2021;16(11):e0259310. doi: 10.1371/journal.pone.0259310 34727140 PMC8562818

[6] Iyoke CA, Lawani OL, Ezugwu EC, Ilechukwu G, Nkwo PO, Mba SG, et al. Prevalence and perinatal mortality associated with preterm births in a tertiary medical center in South East Nigeria. Int J Womens Health. 2014;6:881–8. doi: 10.2147/IJWH.S72229 25378955 PMC4207576

[7] Mini Demographic and Health Survey. 2019. https://www.dhsprogram.com/pubs/pdf/FR363/FR363.pdf.

[8] Abebaw E, Reta A, Kibret GD, Wagnew F. Incidence and Predictors of Mortality among Preterm Neonates Admitted to the Neonatal Intensive Care Unit at Debre Markos Referral Hospital, Northwest Ethiopia. Ethiop J Health Sci. 2021;31(5):937–46. doi: 10.4314/ejhs.v31i5.4 35221609 PMC8843148

[9] Bereka B, Demeke T, Fenta B, Dagnaw Y. Survival status and predictors of mortality among preterm neonates admitted to Mizan Tepi University Teaching Hospital, South West Ethiopia. Pediatric Health, Medicine and Therapeutics. 2021;12:439. 34512074 10.2147/PHMT.S319774 PMC8420788

[10] Desalew A, Sintayehu Y, Teferi N, Amare F, Geda B, Worku T, et al. Cause and predictors of neonatal mortality among neonates admitted to neonatal intensive care units of public hospitals in eastern Ethiopia: a facility-based prospective follow-up study. BMC Pediatr. 2020;20(1):160. doi: 10.1186/s12887-020-02051-7 32290819 PMC7155275

[11] Roba A, Diro D. Morbidities, rate and time trends of neonatal mortality in Dilchora Referral Hospital, Dire Dawa, Ethiopia, 2012-2017. Austin Med Sci. 2017;2(2):1019.

[12] Rhoda N, Velaphi S, Gebhardt G, Kauchali S, Barron P. Reducing neonatal deaths in South Africa: Progress and challenges. South African Medical Journal. 2018;108(3):9–16.

[13] Tolossa T, Fekadu G, Mengist B, Mulisa D, Fetensa G, Bekele D. Impact of antenatal care on neonatal mortality among neonates in Ethiopia: a systematic review and meta-analysis. Arch Public Health. 2020;78(1):114. doi: 10.1186/s13690-020-00499-8 33292564 PMC7653817

[14] Haghighi L, Nojomi M, Mohabbatian B, Najmi Z. Survival predictors of preterm neonates: hospital based study in Iran (2010-2011). Iranian Journal of Reproductive Medicine. 2013;11(12):957. 24639721 PMC3941403

[15] Jain K, Sankar MJ, Nangia S, Ballambattu VB, Sundaram V, Ramji S, et al. Causes of death in preterm neonates (< 33 weeks) born in tertiary care hospitals in India: analysis of three large prospective multicentric cohorts. Journal of Perinatology. 2019;39(1):13–9. 31485016 10.1038/s41372-019-0471-1 PMC8075971

[16] World Health Organization. World health statistics 2016: monitoring health for the SDGs sustainable development goals. World Health Organization. 2016.

[17] Bereka B, Demeke T, Fenta B, Dagnaw Y. Survival Status and Predictors of Mortality Among Preterm Neonates Admitted to Mizan Tepi University Teaching Hospital, South West Ethiopia. Pediatric Health Med Ther. 2021;12:439–49. doi: 10.2147/PHMT.S319774 34512074 PMC8420788

[18] Aynalem YA, Mekonen H, Akalu TY, Gebremichael B, Shiferaw WS. Preterm Neonatal Mortality and its predictors in Tikur Anbessa Specialized Hospital, Addis Ababa, Ethiopia: a retrospective cohort study. Ethiop J Health Sci. 2021;31(1):43–54. doi: 10.4314/ejhs.v31i1.6 34158751 PMC8188116

[19] Yismaw AE, Gelagay AA, Sisay MM. Survival and predictors among preterm neonates admitted at University of Gondar comprehensive specialized hospital neonatal intensive care unit, Northwest Ethiopia. Ital J Pediatr. 2019;45(1):4. doi: 10.1186/s13052-018-0597-3 30616641 PMC6322326

[20] Belay DM, Worku WZ, Wondim A, Hailemeskel HS, Bayih WA. Predictors of survival among preterm neonates admitted to Felege Hiwot comprehensive specialized hospital, Northwest Ethiopia. Frontiers in Pediatrics. 2022;10:196. 10.3389/fped.2022.800300 PMC8965609 35372165

[21] Iyoke CA, Lawani OL, Ezugwu EC, Ilechukwu G, Nkwo PO, Mba SG, et al. Prevalence and perinatal mortality associated with preterm births in a tertiary medical center in South East Nigeria. Int J Womens Health. 2014;6:881–8. doi: 10.2147/IJWH.S72229 25378955 PMC4207576

[22] Mbawala GB, Fredrick F, Kamugisha E, Konje E, Hokororo A. Factors associated with mortality among premature babies admitted at Bugando medical Centre, Mwanza-Tanzania. East Afr J Public Health. 2014;11(1):641–5.

[23] Ballot DE, Chirwa TF, Cooper PA. Determinants of survival in very low birth weight neonates in a public sector hospital in Johannesburg. BMC Pediatr. 2010;10:30. doi: 10.1186/1471-2431-10-30 20444296 PMC2885379

[24] Cupen K, Barran A, Singh V, Dialsingh I. Risk Factors Associated with Preterm Neonatal Mortality: A Case Study Using Data from Mt. Hope Women’s Hospital in Trinidad and Tobago. Children (Basel). 2017;4(12):108. doi: 10.3390/children4120108 29240678 PMC5742753

[25] Yego F, D’Este C, Byles J, Nyongesa P, Williams JS. A case-control study of risk factors for fetal and early neonatal deaths in a tertiary hospital in Kenya. BMC pregnancy and childbirth. 2014;14(1):1–9. 25432735 10.1186/s12884-014-0389-8 PMC4298961

[26] Aslam HM, Saleem S, Afzal R, Iqbal U, Saleem SM, Shaikh MWA, et al. “Risk factors of birth asphyxia”. Ital J Pediatr. 2014;40:94. doi: 10.1186/s13052-014-0094-2 25526846 PMC4300075

[27] Edmond KM, Kirkwood BR, Amenga-Etego S, Owusu-Agyei S, Hurt LS. Effect of early infant feeding practices on infection-specific neonatal mortality: an investigation of the causal links with observational data from rural Ghana. Am J Clin Nutr. 2007;86(4):1126–31. doi: 10.1093/ajcn/86.4.1126 17921392

[28] Jain K, Sankar MJ, Nangia S, Ballambattu VB, Sundaram V, Ramji S, et al. Causes of death in preterm neonates (< 33 weeks) born in tertiary care hospitals in India: analysis of three large prospective multicentric cohorts. Journal of Perinatology. 2019;39(Suppl 1):13–9. 10.1038/s41372-019-0471-1 PMC8075971 31485016

[29] Ghorbani F, Heidarzadeh M, Dastgiri S, Ghazi M, Farshi MR. Survival of premature and low birth weight infants: a multicenter, prospective, cohort study in Iran. Iranian Journal of Neonatology. 2017;8(1).

[30] Cnattingius S, Norman M, Granath F, Petersson G, Stephansson O, Frisell T. Apgar score components at 5 minutes: risks and prediction of neonatal mortality. Paediatric and perinatal epidemiology. 2017;31(4):328–37.\ 28493508 10.1111/ppe.12360

